# Circulating Tumor Cells as a Marker of Disseminated Disease in Patients with Newly Diagnosed High-Risk Prostate Cancer

**DOI:** 10.3390/cancers12010160

**Published:** 2020-01-09

**Authors:** Wojciech A. Cieślikowski, Joanna Budna-Tukan, Monika Świerczewska, Agnieszka Ida, Michał Hrab, Agnieszka Jankowiak, Martine Mazel, Michał Nowicki, Piotr Milecki, Klaus Pantel, Catherine Alix-Panabières, Maciej Zabel, Andrzej Antczak

**Affiliations:** 1 Department of Urology, Poznan University of Medical Sciences, 61-285 Poznan, Poland; agnieszka.ida@gmail.com (A.I.); mhrab@ump.edu.pl (M.H.); aa26@poczta.onet.pl (A.A.); 2 Department of Histology and Embryology, Poznan University of Medical Sciences, 60-781 Poznan, Poland; joanna.budna@wp.pl (J.B.-T.); mswierczewska@ump.edu.pl (M.Ś.); agajankowiak@hotmail.com (A.J.); mnowicki@ump.edu.pl (M.N.); 3 Laboratory of Rare Human Circulating Cells, University Medical Center, 34093 Montpellier CEDEX 5, France; martine-mazel@chu-montpellier.fr (M.M.); c-panabieres@chu-montpellier.fr (C.A.-P.); 4 Department of Electroradiology, Poznan University of Medical Sciences, 61-868 Poznan, Poland; piotrmilecki@ump.edu.pl; 5 Department of Tumor Biology, University Medical Centre Hamburg-Eppendorf, 20246 Hamburg, Germany; pantel@uke.de; 6 Division of Histology and Embryology, Department of Human Morphology and Embryology, Wroclaw Medical University, 50-368 Wroclaw, Poland; mazab@ump.edu.pl; 7 Division of Anatomy and Histology, University of ZielonaGóra, 65-046 ZielonaGóra, Poland

**Keywords:** circulating tumor cells (CTCs), metastases, prostate cancer, radiotherapy, early detection

## Abstract

The aim of this study was to investigate whether the enumeration of circulating tumor cells (CTCs) in blood can differentiate between true localized and metastatic prostate cancer. A cross-sectional study of 104 prostate cancer patients with newly diagnosed high-risk prostate cancer was conducted. In total, 19 patients presented metastatic disease and 85 were diagnosed with localized disease. Analyses included intergroup comparison of CTC counts, determined using the CellSearch^®^ system, EPISPOT assay and GILUPI CellCollector^®^, and ROC analysis verifying the accuracy of CTC count as a maker of disseminated prostate cancer. The vast majority (94.7%) of patients with advanced-stage cancer tested positively for CTCs in at least one of the assays. However, significantly higher CTC counts were determined with the CellSearch^®^ system compared to EPISPOT assay and GILUPI CellCollector^®^. Identification of ≥4 CTCs with the CellSearch^®^ system was the most accurate predictor of metastatic disease (sensitivity 0.500; specificity 0.900; AUC (95% CI) 0.760 (0.613–0.908). Furthermore, we tried to create a model to enhance the specificity and sensitivity of metastatic prediction with CTC counts by incorporating patient’s clinical data, including PSA serum levels, Gleason score and clinical stage. The composite biomarker panel achieved the following performance: sensitivity, 0.611; specificity, 0.971; AUC (95% CI), 0.901 (0.810–0.993). Thus, although the sensitivity of CTC detection needs to be further increased, our findings suggest that high CTC counts might contribute to the identification of high-risk prostate cancer patients with occult metastases at the time of diagnosis.

## 1. Introduction

Prostate cancer is one of the most common male malignancies in industrialized countries [1]. According to the most recent data, the 5-year relative survival rate of prostate cancer patients in Europe is 82% [2]. Such favorable prognosis results from the fact that the vast majority of currently detected prostate cancers are low- or intermediate-risk tumors with a low likelihood of progression [3]. However, the population of prostate cancer patients also includes ca. 15–20% of subjects with high-risk malignancies associated with a very high (ca. 80%) likelihood of systemic spread, primarily to the bones [4]. Importantly, even up to 20% of prostate cancers may be disseminated already at the time of primary diagnosis [5]. Prognosis in such cases is poor, with 5-year relative survival rates markedly lower than in the general population of prostate cancer patients [6]. Unfortunately, none of the currently available scoring systems is not accurate enough to identify patients with a high risk of prostate cancer spread and/or occult disseminated disease [7].

This justifies research on a novel method enabling identification of high-risk patients and covering them with an active surveillance. Available evidence suggests that an ideal candidate for such a method is enumeration of circulating tumor cells (CTCs) in patients’ blood. CTCs were first found in peripheral blood nearly 150 years ago, in 1869 [8]. They represent a specific subset of cancer cells that, due to epithelial-to-mesenchymal transition (EMT), acquired the ability to leave the primary tumor, intravasate into circulation, survive and proliferate in a new location [9]. Until recently, our ability to isolate CTCs from peripheral blood was limited due to their rarity [10]. However, identification of specific morphological and antigenic characteristics of CTCs resulted in the development of novel enrichment and isolation methods. The majority of these methods is based on immunoaffinity and utilizes positive or negative enrichment. The first approach enables selection of CTCs by labeling tumor-associated antigens. Its widely used types include immunomagnetic assays, i.e., CellSearch^®^ [11], MACS [12], and Adna Test [13], microfluidic chips, i.e., multi-flow microfluidic (MFM) system [14], Herringbone Chip [15], and spiral ClearCell^®^ FX chip [16], as well as combined solutions, like IsoFlux [17], LiquidBiopsy [18] or Ephesia [19]. Among them, the CellSearch^®^ system, based on detection of a cell surface marker of CTCs, epithelial cell adhesion molecule (EpCAM), staining with 4′,6-diamidino-2-phenylindole (DAPI) nuclear stain and antibodies against cytokeratins and CD45, has already been cleared by the US Food and Drug Administration for the detection of circulating tumor cells in metastatic breast, colon and prostate cancer [20,21]. Two other technologies for CTC enumeration, the EPISPOT assay and CellCollector^®^, have been developed and are currently tested for their non-inferiority or complementarity to the CellSearch^®^ platform [22,23]. On the contrary, negative enrichment technologies, like QMS [24] or EasyStep Human CD45 Depletion Kit [25], implement antibodies against leukocyte-associated antigens.

Apart from EpCAM and cancer-specific antigens, currently developed CTC detection methods permit recognition of other CTC biomarkers of great clinical impact, such as programmed cell death ligand 1 (PD-L1). Its ability to restrain an anti-cancer immune response is the vital cause of therapy failure. Thus, enrichment of standard CTC detection with simultaneous analysis of PD-L1 expression was found to be beneficial for assessing the effectiveness of immune therapies and risk of resistance development in head and neck squamous cell carcinoma [26], metastatic breast cancer [27] and lung cancer [28].

The results of previous studies verifying the prognostic value of CTC counts in advanced metastatic prostate cancer are highly promising. Lower baseline counts of CTCs determined with aid of the CellSearch^®^ system, and/or their reduction after chemotherapy were shown to be associated with a survival benefit [20,29,30,31,32,33]. However, to the best of our knowledge, none of the previous studies verified another potential application of CTC enumeration, i.e., the differentiation of true localized prostate cancer from occult disseminated disease. Having access to a relatively large cohort of patients with newly diagnosed high-risk prostate cancer, including a subset of subjects presenting with confirmed disseminated disease and a subgroup with truly localized malignancy, we investigated if CTC counts determined using the CellSearch^®^ system, EPISPOT assay and CellCollector^®^ may accurately differentiate between localized and metastatic prostate cancer. We hypothesized that patients with metastatic prostate cancer are more likely to test positively for CTCs with higher CTC counts with CellSearch^®^. Thus, we assumed that due to different approaches to enrich and detect CTCs, these three technologies may detect different sub-populations of CTCs and hence provide complementary results, increasing the sensitivity of CTC detection.

## 2. Results

### 2.1. Patient Characteristics

The study included 104 prostate cancer patients with a mean age of 68.2 ± 6.5 years (range 51–86 years). Other characteristics of the study subjects are presented in Table 1.

### 2.2. CTC Counts Obtained with Three Enumeration Technologies

**Figure 1:**
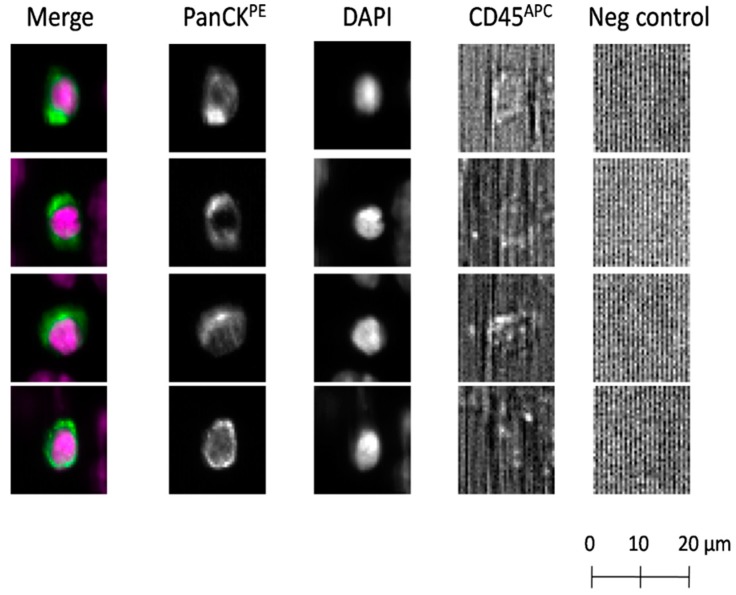
Detection of circulating tumor cells (CTCs) using the CellSearch^®^ system. CTCs identified according to the following criteria: EpCAM-positive, panCK-positive, DAPI-positive, CD45-negative and negative for the last channel. CK: cytokeratin; panCK: anti-CK8, -18, -19 antibodies; PE: phycoerythrin; APC: allophycocyanin.

**Figure 2:**
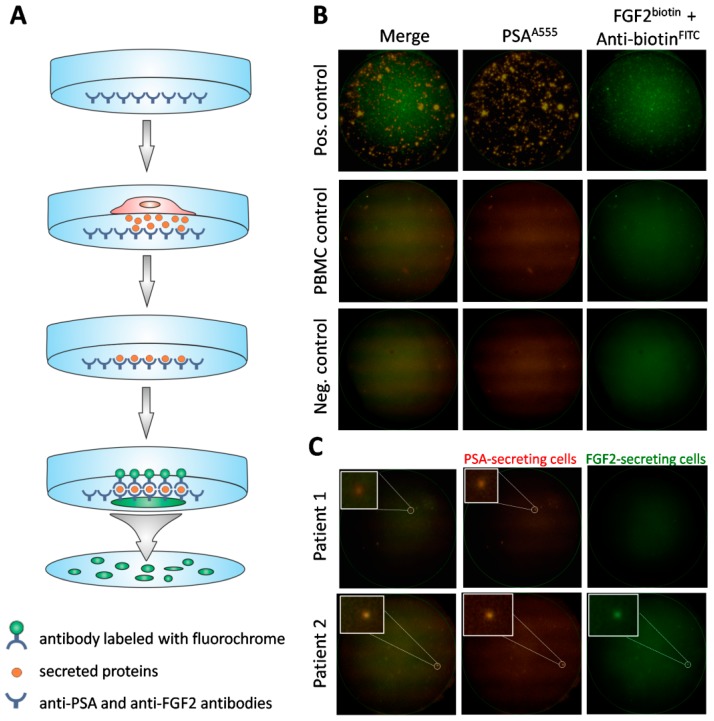
Detection of viable CTCs using the dual fluorescent EPISPOT^PSA/FGF2^ assay. (**A**) Procedure of the assay. Membrane covered with anti-PSA and anti-FGF2 antibodies, culture of cells enriched via depletion of CD45^+^ cells, binding of secreted proteins to previously immobilized antibodies, immunostaining of secreted proteins caught on the membrane. (**B**) Positive and negative controls. Culture of LNCaP (shown in figure) and NBTII cells (secreting PSA and FGF2, respectively) as positive controls, culture of PBMC (not secreting PSA nor FGF2) and wells without cells used as negative controls. A single immunospot corresponds to the protein “fingerprint” of one viable cell. (**C**) Patient samples. Representative examples of CTCs defined based on PSA-secretion (Patient 1) or PSA and additionally, FGF2 (Patient 2). PSA-positive, FGF2-positive and double PSA/FGF2 immunospots (merge) correspond to viable CTCs. Immunospots were detected and observed using the C.T.L. Elispot Reader, 50× magnification.

**Figure 3:**
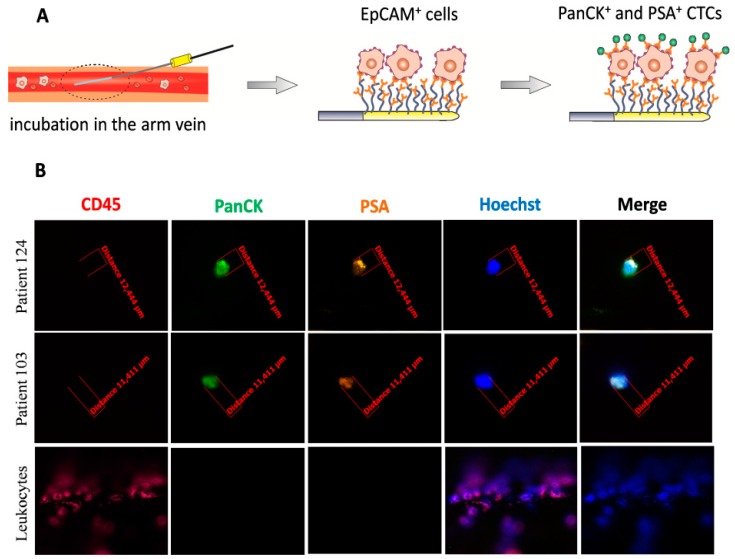
Detection of CTCs captured by the CellCollector^®^ in vivo system. (**A**) Procedure of the CellCollector^®^ detection. After 30 min incubation in patient’s arm vein, removal of the device together with captured EpCAM^+^ cells, immunostaining of isolated cells with anti-panCK, anti-PSA, anti-CD45 antibodies and Hoechst nuclear staining for detection of CTCs. (**B**) Representative images of CTCs isolated in vivo and non-specifically bound leukocytes (CD45-positive). CTCs were identified according to the following criteria: panCK-positive (green), CD45-negative (red) and optionally, PSA-positive (orange), nucleus stained with Hoechst 33258 (blue). Images were obtained using a fluorescent microscope (Carl Zeiss, Axio Imager 2, 20×) and analyzed with the Carl Zeiss, Axio Vision 4.8 software.

Using the CellCollector^®^, CTCs were found in 60/104 (57.7%) patients, with the aid of the dual fluoro-EPISPOT^PSA/FGF2^ assay in 52/100 (52.0%), and by means of the CellSearch^®^ system in 21/88 (23.9%). Representative examples of positive CTC signals for each of three tested technologies were presented on Figure 1, Figure 2B and Figure 3B, respectively.

Statistical characteristics of CTC counts determined with each technique are presented in Table 2.

No statistically significant correlations were found between the number of CTCs detected with the CellCollector^®^ and the results obtained with either the dual fluoro-EPISPOT^PSA/FGF2^ assay (*R* = −0.001, *p* = 0.990) or the CellSearch^®^ system (*R* = −0.031, *p* = 0.772). However, a weak inverse correlation was observed between the results obtained with the dual fluoro-EPISPOT^PSA/FGF2^ and the CellSearch^®^ system (*R* = −0.215, *p* = 0.049). Contingency of positive/negative results obtained with each technique was very low, Φ = 0.015 for the CellCollector^®^ and the dual fluoro-EPISPOT^PSA/FGF2^, Φ = −0.090 for the CellCollector^®^ and detection with the CellSearch^®^ system, and Φ = −0.225 for the dual fluoro-EPISPOT^PSA/FGF2^ assay and detection with the CellSearch^®^ system (Table 3).

CTC counts determined with the CellCollector^®^, dual fluoro-EPISPOT^PSA/FGF2^ assay and CellSearch^®^ system did not correlate significantly with the age of the study subjects, PSA concentration and the number of satisfied D’Amico criteria. Biopsy Gleason sum correlated significantly with the number of CTCs determined with the CellSearch^®^ system, but not with the results obtained with the CellCollector^®^ and dual fluoro-EPISPOT^PSA/FGF2^ assay (Supplementary Table S1). In line with these findings, the proportion of patients with biopsy Gleason sum ≥8 pts turned out to be significantly higher in the group with the positive result of testing with the CellSearch^®^ system than in that without. Groups with positive and negative results obtained with the CellCollector^®^ and dual fluoro-EPISPOT^PSA/FGF2^ assay did not differ significantly in the proportions of individuals with biopsy Gleason sums of 8 pts and more. Irrespective of the technique used to detect CTCs, groups with positive and negative results of the assay contained similar proportions of subjects with PSA concentrations >20 ng/mL (Supplementary Table S2).

Distant metastases were found in 19 (18.3%) patients; 17 of these patients had isolated or concomitant bone metastases. Although the vast majority (18/19, 94.7%) of patients with metastatic prostate cancer tested positively for CTCs when examined with the CellCollector^®^ and/or dual fluoro-EPISPOT^PSA/FGF2^ assay and/or CellSearch^®^ system, an equally large proportion of the positive results was documented in the non-metastatic group (Table 4).

Diagnostic accuracy of all factors showing statistically significant associations with the presence of distant metastases was verified using ROC analysis (Table 5).

**Figure 4:**
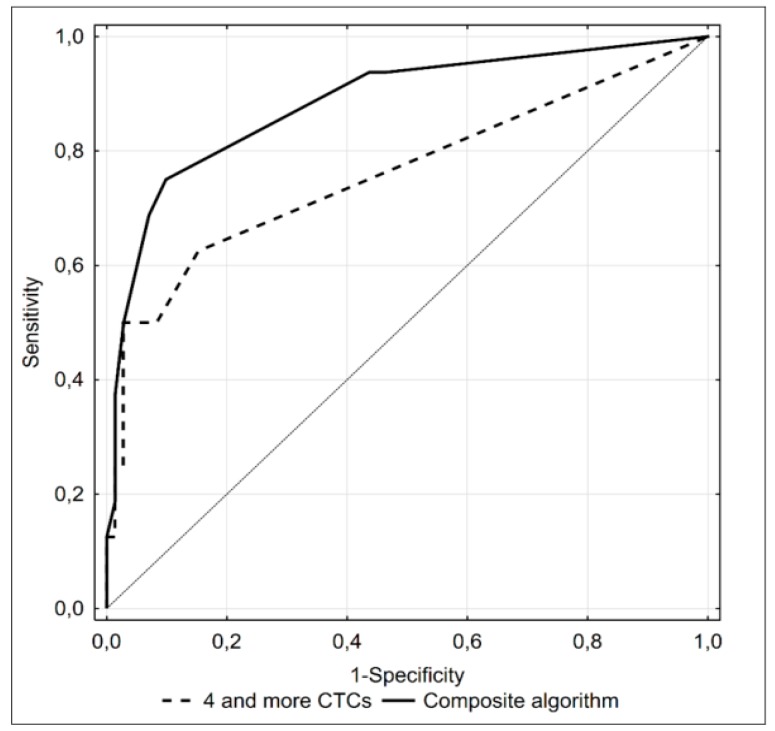
ROC curve illustrating diagnostic accuracy of ≥4 CTCs detected with the CellSearch^®^ system (dotted line) and a composite algorithm including this parameter, concentration of PSA >25 ng/mL, Gleason score equal to 9 points and cT > 2C as predictors of bone metastases (black line) in prostate cancer patients.

Presence of at least 4 CTCs turned out to be the most accurate predictor, providing 9/10 true positive and 69/78 true negative results. However, the AUC under the ROC curve for the CTC count was still relatively small. Therefore, we looked for a composite predictive algorithm with a higher diagnostic accuracy. Aside from a CTC count ≥ 4, the composite model included a concentration of PSA >25 ng/mL, biopsy Gleason sum equal to 9 pts and cT > 2c; we did not include the number of satisfied D’Amico criteria in the composite algorithm as this parameter was an equivalent for the abovementioned three variables. None of 19 subjects with distant metastases satisfied all four criteria of the model (Supplementary Table S3). Nevertheless, inclusion of an additional three variables significantly increased the accuracy of the diagnostic algorithm (Figure 4), providing 11/13 true positive and 67/74 true negative results.

## 3. Discussion

Our series included 19 patients with high-risk prostate cancer, who presented with metastatic disease, preferentially bone metastases (17/19), at the time of diagnosis. Individuals with distant metastases presented with significantly higher CTC counts determined with the CellSearch^®^ system and significantly more often tested positively for CTCs when examined with this assay. Moreover, the group with metastases was characterized by significantly higher median values for PSA concentration, biopsy Gleason sums and the number of satisfied D’Amico criteria, and included significantly larger proportions of subjects with Gleason sums ≥ 8 pts and cT > 2C. When examined with the CellSearch^®^ system, these subjects tested positively for CTCs significantly more often and presented significantly higher CTC counts than other individuals with high-risk prostate cancer. Moreover, in this study, we validated CTC counts by two other methods CellCollector^®^ and dual fluoro-EPISPOT^PSA/FGF2^ assay. In a previous study conducted by Danila et al. [29], who analyzed prognostic value of CTC count in 120 subjects with progressive castration-resistant prostate cancer using CellSearch^®^ system, median CTC counts in patients with bone metastases alone or with bone and soft tissue involvement were 10.5 or 13.5 cells, respectively, as compared to 2.5 cells in those with non-skeletal spread alone [29]. Our study only included two patients with isolated soft tissue metastases. One patient tested negatively for CTCs when examined with the CellSearch^®^ system, while the other one presented with 12 CTCs. However, even after exclusion of these subjects, CTC counts determined with the CellSearch^®^ system in the remaining subset of 17 patients with bone metastases (median 2.5 cells) were 4- to 5-fold lower than in those examined by Danila et al. [29]. There are several potential explanations for this discrepancy. The series examined by Danila et al. [29] included a subgroup of patients with previous history of a hormone therapy and cytotoxic chemotherapy who were already castration resistant, and these subjects presented with significantly higher CTC counts due to the more advanced stage of disease. In contrast, our series was comprised solely of treatment-naive individuals with newly diagnosed prostate cancer.

To complement the results obtained with the CellSearch^®^ system, we used two other techniques for CTC enumeration—dual-fluoro-EPISPOT^PSA/FGF2^ assay and CellCollector^®^ CANCER01 (GILUPI GmbH). EPISPOT assay is based on the detection of proteins secreted by functional (viable) CTCs, combined with a negative enrichment (leukocyte depletion) [34]. Another recently developed CTC assay is the CellCollector^®^, a device that captures CTCs in vivo, in the peripheral arm vein, based on their affinity to anti-EpCAM antibodies [33,35]. Considering their different mechanisms and targets for CTC capture, we expected that these three techniques for CTC enumeration may produce complementary results. Indeed, we found that the correlations between absolute CTC counts or contingency of positive/negative results obtained with these techniques were very low. Furthermore, 18 of 19 patients who presented with disseminated prostate cancer at the time of diagnosis had CTCs in at least one of these three assays. This observation is consistent with the results of a previous study, in which the CellSearch^®^ system and EPISPOT assay were analyzed together as predictors of overall survival in metastatic breast cancer patients; although the overlap of the results obtained with both techniques was low (*d* = 0.14), their combination turned out be the strongest predictor of overall survival in multivariate analysis [34].

Interestingly, positive results of at least one out of three CTC assays were also obtained in 72/85 (84.7%) patients with no evidence of tumor spread in imaging studies. According to the literature, around 80% of patients with high-risk prostate cancer may develop distant metastases during follow-up [3]. Therefore, it can be hypothesized that a considerable proportion of 72 CTC-positive and metastasis-negative patients included in our series presented with occult disseminated disease; ongoing follow-up study of these subjects will soon explain whether this hypothesis was true or not. Nevertheless, our findings suggest that all patients with newly diagnosed high-risk prostate cancer, who tested positively for CTCs in the triple assay, should be screened repeatedly for metastasis.

In the current study, all CTCs were detected as single cells. Aceto et al. [36,37] documented that the presence of CTC clusters is associated with an unfavorable prognosis in patients with diverse tumors. Compared to clusters, single CTCs in the bloodstream are more prone to undergo anoikis, programmed cell death caused by loss of cell–extracellular matrix interactions. CTC groups are more likely to form metastases, since their tight junctions increase apoptosis resistance. Studies conducted on CTCs clusters in metastatic breast cancer patients revealed the presence of hypomethylated binding sites for transcription factors associated with stemness and proliferation, favoring tumor spread [36]. Furthermore, clusters of CTCs associated with white blood cells, predominantly neutrophils, were found to promote cell proliferation in the bloodstream and hence, metastasis in breast cancer [37]. The lack of CTC clusters, characteristic of metastatic cancers, in our study can possibly be attributed to a relatively small subset of metastatic patients.

Despite the excellent detection rate for disseminated disease, a positive result of the triple CTC assay did not differentiate the subjects with and without metastatic prostate cancer. Therefore, we developed a composite algorithm including clinical parameters whose values differed significantly between the metastatic and non-metastatic group. As already mentioned, these two groups differed in terms of their CTC counts determined with the CellSearch^®^ assay, and ≥4 CTCs turned out to be the cut-off value which most accurately identified subjects with disseminated disease in ROC analysis. This cut-off value was similar to those identified in previous studies examining predictive values of the CTC counts determined with the CellSearch^®^ system, whereby survival benefit was associated with CTC < 4 or < 5 [20,30,31,32,33].

However, considering these results in terms of clinical relevance, one must keep in mind that the population of CTCs derived from the same tumor can be highly heterogenous. Intratumoral heterogeneity (ITH) includes genetic, imunophenotypic and even functional variations [38]. Mutations in DNA control and repair genes can cause the formation of distinct cancer clones [39] of different tumor biology, influencing the effectiveness of invasion and metastasis formation, as well as drug resistance [40]. To asses tumor heterogeneity and estimate premetastatic potential of CTCs, single-cell isolation and sequencing studies can be performed. In-depth analysis of PD-L1 expression [41], stemness [42], drug resistance [43] and EMT [44] can help to undertake treatment decisions and foresee clinical impact. The EMT process is most often regarded as a cause of CTC detection failure since expression of epithelial markers is diminished, causing limited usefulness of EpCAM- and cytokeratin-based technologies, like CellSearch^®^ [45]. As a result, the non-epithelial subpopulation of CTCs can be omitted during the selection step [46]. The test is challenging due to a low recovery rate, which can be associated with a high level of apoptosis among circulating cells. On the other hand, the evaluation of apoptosis among these cells is extremely informative, since a low predominance of apoptotic cells has already been related to poor prognosis and aggressive phenotypes in cancers [47].

Gupta et al. [48] presented results that are in line with the above statements. A paired comparison of genomic alterations between CTCs and cfDNA in metastatic castration-resistant prostate cancer, showed both concordance and disconcordance of certain alterations. The results enabled the prognosis of the clinical outcome for certain relations to be specified and proved that tumor heterogeneity is strictly linked to poor clinical outcomes [48].

Additionally, our team performed molecular analysis of CTCs captured by CellCollector^®^. RT-qPCR multiplex analysis of mRNA coding EMT, epithelial, and stem cell markers showed a predominant expression of EMT-associated markers. CTCs presenting such a phenotype would be lost if selected solely based on epithelial markers, thus this outcome underlined the need of the application of various antibodies to effectively capture different CTCs phenotypes [49].

In the current study, in addition to CTC counts, we also identified other significant predictors of metastatic prostate cancer: PSA > 25 ng/mL, Gleason sum of at least 9 pts, cT > 2c and presence of all three D’Amico criteria. Two of these parameters (PSA and Gleason score), albeit with different cut-off values, are included in the algorithm and are proposed as prognostic markers by the European Association of Urology (EAU) [7,50]. Our present findings show high NPVs for both of these parameters, similar to that for the CTC count threshold of ≥4. However, the PPVs for PSA concentration and Gleason score were markedly lower than the PPV for the CTC count, which implies that the two established parameters are more suitable to exclude disseminated disease than CTC counts, but still lack adequate sensitivity. Indeed, inclusion of PSA > 25 ng/mL, Gleason scores of ≥9, cT > 2c and CTC count ≥ 4 in the composite diagnostic algorithm did not improve its PPV over that obtained with the CTC number alone, despite a significant increase in AUC; this finding suggests that a CTC count ≥ 4 may be considered as an independent marker of systemic spread in newly diagnosed high-risk prostate cancer.

Although the hereby presented findings seem promising from a clinical perspective, we are well aware of the potential limitations of our study. Firstly, the subset of patients with newly diagnosed high-risk prostate cancer was quite small. Secondly, we should consider the limited sensitivity of the reference methods (computed tomography and radionuclide bone scan) used to confirm the presence of metastatic disease [7]. Furthermore, we still await the follow-up data of patients who tested positively for CTCs despite the lack of clinical evidence of a systemic spread. Additionally, a growing body of evidence suggests that some CTCs can undergone EMT and these cells might have down-regulated expression of EpCAM, a key molecule used to enrich CTCs in the CellSearch^®^ system [51]. EpCAM-selectivity also significantly limits the effectiveness of cancer stem cell detection. These undifferentiated cells are predominantly linked to the process of colonization in the invasion-metastasis cascade, constituting a major step of tumor outgrowth [45]. In addition, the number of CTCs in a single 7.5 mL blood sample may not necessarily reflect their true count in total blood due to the small sample volume [10]. Nevertheless, CellSearch^®^ remains the most widely validated CTC detection technology and has been cleared by the Food and Drug Administration for CTC counting in metastatic cancers. Furthermore, the prognostic relevance of CTCs detected with CellSearch^®^ has been proven in breast, colorectal, esophagus, bladder and prostate cancers [20,21].

Recent evidence suggests that CTCs are highly heterogeneous in terms of their molecular characteristics and metastatic potential [52]. While application of the CellSearch^®^ system allows molecular typing of single CTCs [22,23,53,54], tumor cells captured with EPISPOT assay and CellCollector^®^ seem to be more suitable for this type of analysis. Although CTCs cannot be removed from the CellCollector^®^, it is possible to cut the wire and analyze the CTC-enriched fraction using RT-PCR [49]. At present, the EPISPOT assay is further developed into a new format—called EPIDROP—that will allow downstream analyses of captured CTCs [55]. Future research should, therefore, include the molecular analysis of CTCs to increase diagnostic accuracy and obtain information on therapeutic targets and resistance mechanisms [55,56].

## 4. Materials and Methods

### 4.1. Patients

The material for the study was collected at the Greater Poland Cancer Center in Poznan, within the framework of the international multicenter project ERA-NET-TRANSCAN entitled “Circulating Tumor Cells as Biomarker for Minimal Residual Disease in Prostate Cancer” (acronym: CTC-SCAN). The aim of the CTC-SCAN project was to validate the number of CTCs isolated from patient’s blood as a prognostic marker for relapse in high-risk prostate cancer treated with primary radical prostatectomy or radiotherapy. Only patients with newly diagnosed non-metastatic prostate cancer, representing a high-risk group according to the D’Amico criteria (cT ≥ 2c and/or PSA ≥ 20 ng/mL and/or biopsy Gleason sum ≥8) [57], were eligible for the CTC-SCAN project. However, a considerable proportion of patients enrolled in our center were diagnosed with disseminated disease at the time of diagnosis. Hence, this subgroup, along with the remaining participants with truly localized prostate cancer, qualified for this satellite study.

### 4.2. Ethics

Protocol of the study was approved by the Local Bioethical Committee at the Poznan University of Medical Sciences (decision no. 28/13 of 3 January 2013), with written informed consent sought from all the study subjects.

### 4.3. Diagnosis and Staging

Diagnosis of prostate cancer was established on the basis of history taking, physical examination, measurement of serum PSA, and 10–12 core needle biopsies with the determination of biopsy Gleason sum. To exclude soft tissue disease, abdominal and pelvic computed tomography scans were reviewed. Moreover, radionuclide bone scans were evaluated for the presence or absence of metastatic bone disease. The stage of the disease was defined according to the 7th edition of The American Joint Committee on Cancer (AJCC) staging manual [58].

### 4.4. CTC Enumeration

After establishing the diagnosis, prior to implementation of any anti-cancer treatment, CTCs were enumerated using three different assay formats: CellSearch^®^ system (Silicon Biosystem, Menarini, Bologna, Italy), dual fluoro-EPISPOT^PSA/FGF2^ assay and CellCollector^®^ CANCER01 (GILUPI GmbH, Potsdam, Germany). For the CellSearch^®^ system, 7.5 mL of blood was drawn into CellSave^®^ tubes (Silicon Biosystem, Menarini) and sent at room temperature on the same day to the Laboratory of Rare Human Circulating Cells (LCCRH, Montpellier, France) at the University Medical Center of Montpellier, France, where analysis took place on the day of arrival. For the dual fluoro-EPISPOT^PSA/FGF2^ assay 10 mL of blood was collected in EDTA tubes and processed on the same day. Finally, for CTC isolation with CellCollector^®^ CANCER01 (GILUPI GmbH), the device was inserted into the patient’s arm vein for 30 min. Enumeration with both the dual fluoro-EPISPOT^PSA/FGF2^ assay and CellCollector^®^ CANCER01 were carried out in the Department of Histology and Embryology at the Poznan University of Medical Sciences, Poland.

#### 4.4.1. CellSearch^®^ System

A 7.5 mL of venous blood collected to the CellSave^®^ tube (Silicon Biosystem, Menarini, Bologna, Italy) was analyzed and CTCs were enumerated with the use of Circulating Epithelial Cell Kit (Silicon Biosystems, Menarini, Bologna, Italy). The system enables enrichment of CTCs with magnetic beads coated with anti-EpCAM antibodies carried by ferrofluid. Captured target cells were then immunostained with antibodies against cytokeratins (panCK = CK8, 18 and 19), and the common leukocyte antigen CD45 to exclude leukocytes. Cells positive for EpCAM, cytokeratins with positive DAPI staining as a measure of nuclear integrity, and negative for CD45, were identified as CTCs.

#### 4.4.2. Dual Fluoro-EPISPOT^PSA/FGF2^ Assay

A 10 mL venous blood sample was obtained from each patient and drawn into an EDTA-coated tube. CTCs were enumerated and characterized using the dual fluorescent EPISPOT^PSA/FGF2^ assay. During the first step, CD45-positive cells were depleted from the sample using 50 µL of RosetteSep^TM^ Human Circulating Epithelial Tumor Cell Enrichment Cocktail (STEMCELL Technologies, Vancouver, Canada) per 1 mL of blood. After incubation, according to the manufacturer’s protocol, cells were subjected to phase separation with a 1.073 density gradient, collected from the interphase and washed twice. Subsequently, the CD45-depleted cell fraction was used for the proper dual fluoro-EPISPOT^PSA/FGF2^ assay. Briefly, the nitrocellulose membranes of the EPISPOT plates were coated with 1.04 µg/µL of anti-PSA H50 antibody (obtained from the Department of Biotechnology, University of Turku, Turku, Finland) and 0.5 µg/µL of anti-FGF2 500-M38 antibody (Peprotech, London, UK) diluted in PBS and blocked with 5% BSA/PBS. Then, cells were seeded in each well and cultured for 48 h at 37 °C and 5% CO_2_. During this incubation step, the secreted marker proteins were directly captured on the antibody-coated membrane. Next, cells were washed off and the marker proteins were detected by secondary antibodies conjugated with fluorochrome dyes: 1.0 µg/µL of anti-PSA-H117-A555 antibody (obtained from the Department of Biotechnology, University of Turku, Turku, Finland) and 0.5 µg/µL of anti-FGF2 500-P18Bt labeled with biotin (Peprotech) and subsequently with 1:20 anti-biotin-FITC antibody (MiltenyiBiotec, Bergisch Gladbach, Germany) diluted in 0.5% BSA/PBS. After washing, PSA and FGF2 immunospots were counted under a fluorescent microscope by video camera imaging and computer-assisted analysis (KS ELISPOT, Carl Zeiss Vision, Oberkochen, Germany) and the C.T.L. ELISPOT reader (Autoimmun Diagnostika, Strassberg, Germany): one immunospot corresponded to the fingerprint of one viable marker protein-secreting cell. For positive control for PSA and FGF2 proteins, LNCap and NBTII cell lines were used, respectively. LNCaP cells (ATCC CRL-1740) were cultured in RPMI 1640 medium (Thermo Fisher Scientific, Waltham, MA, USA), while NBTII cells (ATCC CRL-1655) were cultured in DMEM medium with GlutaMAX (Gibco), both were supplemented with 10% fetal bovine serum (FBS) (Sigma Aldrich, Merck, Darmstadt, Germany) and antibiotics (Sigma Aldrich). Cells were cultured in 75 cm^2^ flasks and, upon reaching confluence of 80%, detached (0.25% trypsin/EDTA, Gibco) and were washed and counted. A total of 2000 cells/well (two wells) were seeded in each plate. Then, as the next step of the dual fluoro-EPISPOT^PSA/FGF2^ procedure, enriched CTCs, LNCap and NBTII cells were cultured in RPMI 1640 medium (Sigma Aldrich) supplemented with 10% FBS (Sigma Aldrich), antibiotics (Sigma Aldrich), 1% l-glutamine (Sigma Aldrich) and 1% Insulin-Transferrin-Selenium (Gibco). The procedure of dual fluoro-EPISPOT^PSA/FGF2^ assay is presented in Figure 2A.

All assays were conducted at the Department of Histology and Embryology, Poznan University of Medical Sciences, and then, their results were verified and validated at the Laboratory of Rare Human Circulating Cells, University Medical Center in Montpellier (Montpellier, France).

#### 4.4.3. CellCollector^®^ CANCER01 (GILUPI GmbH)

CellCollector^®^ was inserted into the patient’s arm vein via a standard 20-gauge needle. During the 30 min application into the vein, up to 1500 mL of blood including the respective CTCs passed the 2 cm functionalized area of the CellCollector^®^. Passing CTCs were bound by the anti-EpCAM antibody and removed from the patient’s vein together with the CellCollector^®^. Then, cells captured on the wire were fixed in cold acetone for 10 min, permeabilized in 0.1% Triton X-100/PBS for 10 min, and blocked in 3% BSA/PBS for 30 min. Fixed cells were characterized by immunostaining with fluorochrome-labeled anti-cytokeratin antibodies (both 1:50, anti-panCK-A488, eBiosience; anti-panCK-A488, Exbio, Thermo Fisher Scientific, Waltham, MA, USA), anti-PSA antibody (1:80, anti-PSA-H117-A555 antibody; obtained from the Department of Biotechnology, University of Turku, Turku, Finland), an antibody against the leukocyte marker CD45 (1:25, CD45-A647, Exbio), as well as a staining of the nucleus (1 µg/mL, Hoechst 33258, Sigma), all diluted in 3% BSA/PBS. The antibodies were conjugated with different fluorescent dyes, allowing discrimination between CTCs and leukocytes by fluorescence microscopy. Images were taken with the use of a fluorescent microscope (Axio Imager 2, Carl Zeiss, Oberkochen, Germany), 20× objective and were analyzed with AxioVision 4.8 (Carl Zeiss, Oberkochen, Germany). CTCs were characterized as cytokeratin-positive, and/or PSA-positive and CD45-negative nucleated cells with an intact morphology and diameter ≥4 µm. The procedure of CTCs isolation using CellCollector^®^ is presented in Figure 3A.

### 4.5. Statistical Analysis

Normal distribution of continuous variables was verified with a Shapiro–Wilk test. Statistical characteristics of continuous variables are presented as means, standard deviations (SD), medians and ranges. The significance of intergroup differences in the characteristics of continuous variables was verified with a Mann–Whitney *U*-test. Power and direction of associations between pairs of continuous variables were estimated on the basis of Spearman’s rank correlation coefficients (R). Statistical characteristics of discrete variables are presented as numbers and percentages and compared between the groups using Pearson’s chi-square test or Fisher’s exact test. Power and direction of associations between distributions of two discrete variables were estimated on the basis of contingency coefficients (Φ). The variables that showed significant associations with the presence of disseminated disease on univariate analysis were subjected to receiver operating characteristic (ROC) analysis. In the case of continuous variables, their cut-off values, characterized by the lowest error rate, were determined. Sensitivity, specificity, and positive and negative predictive value (PPV and NPV) for these cut-off values were calculated, as well as the areas under ROC curves (AUC) with 95% CI. Moreover, the diagnostic accuracy of composite algorithms including more than one predictor of disseminated disease was tested on ROC analysis; in such cases, expected values from multivariate logistic regression analysis were analyzed. AUC values for single explanatory variables and combinations thereof were compared with a Z-test. All calculations were carried out using Statistica 10 package (StatSoft, Dell, Round Rock, TX, USA), with the threshold of statistical significance set at *p* ≤ 0.05.

## 5. Conclusions

The presence of ≥4 CTCs determined with the CellSearch^®^ system may be considered as marker of metastatic high-risk prostate cancer that provides additional information to the current risk score. Future follow-up studies will show whether detection of lower CTC counts by CellSearch^®^ alone or together with the CellCollector^®^ and/or EPISPOT assay can identify patients with occult disseminated disease.

## Figures and Tables

**Table 1:** Clinical characteristics of prostate cancer patients participating in the study (*n* = 104).

Parameter	Median (Range)/*n* (%)
PSA (ng/mL)	29.5 (0.5–191.0)
PSA > 20 ng/mL (*n*)	81 (77.9%)
Gleason sum (pts)	7 (6–9)
Gleason sum ≥ 8 pts (*n*)	29 (27.9%)
cT > 2c (*n*)	48 (46.6%)
D’Amico criteria (*n*)	1 (1–3)

PSA—Prostate Specific Antigen.

**Table 2:** Median number of CTCs detected using the CellCollector^®^, dual fluoro-EPISPOT^PSA/FGF2^ assay and CellSearch^®^ system.

Method	Median (Range)
CellCollector^®^ (*n*)	1 (0–7)
EPISPOT assay (*n*)	1 (0–25)
CellSearch^®^ system (*n*)	0 (0–569)

**Table 3:** Contingency table illustrating distribution of positive (at least one CTC in analyzed material) and negative (no CTCs) results obtained with the CellCollector^®^, dual fluoro-EPISPOT^PSA/FGF2^ assay and CellSearch^®^ system; consistent results highlighted in gray.

Assay		CellCollector^®^		EPISPOT Assay	
		(+)	(−)	(+)	(−)
EPISPOT assay	(+)	30/100 (30.0%)	22/100 (22.0%)		
	(−)	27/100 (27.0%)	21/100 (21.0%)		
CellSearch^®^ system	(+)	11/88 (12.5%)	10/88 (11.4%)	6/84 (7.1%)	13/84 (15.5%)
	(−)	42/88 (47.7%)	25/88 (28.4%)	38/84 (45.2%)	27/84 (32.1%)

**Table 4:** Characteristics of patients with and without distant metastases of prostate cancer.

Parameter	Metastases (*n* = 19)	Local Tumor (*n* = 85)	*p*-Value
Number of CTCs (*n*):			
CellCollector^®^	1 (0–5)	1 (0–7)	0.524
EPISPOT assay	0 (0–2)	1 (0–25)	0.116
CellSearch^®^ system	2.5 (0–569)	0 (0–54)	<0.001
Positive results (*n*):			
CellCollector^®^ (I)	10 (52.6%)	50 (58.8%)	0.619
EPISPOT assay (II)	7 (38.9%) ^1^	45 (54.9%) ^2^	0.299
CellSearch^®^ system (III)	11 (61.1%) ^1^	10 (14.3%) ^3^	<0.001
I + II + III	18 (94.7%)	72 (84.7%)	0.457
Age (years)	67.8 ± 6.8	68.3 ± 6.4	0.791
PSA (ng/mL)	53.0 (0.6–164.0)	28.4 (0.5–191.0)	0.022
PSA > 20 ng/mL (*n*)	15 (78.9%)	66 (77.6%)	0.902
Gleason sum (pts)	8 (6–9)	7 (6–9)	0.004
Gleason sum ≥ 8 pts (*n*)	10 (52.6%)	19 (22.4%)	0.012
cT > c (*n*)	14 (73.7%)	34 (40.5%) ^4^	0.011
D’Amico criteria (*n*)	2 (1–3)	1 (1–3)	<0.001

Different group size than specified in the column header: ^1^ *n* = 18, ^2^ *n* = 82, ^3^ *n* = 70, ^4^ *n* = 84.

**Table 5:** Significant predictors of concomitant distant metastases in prostate cancer patients—results of ROC analysis.

Parameter	Sensitivity	Specificity	PPV	NPV	AUC (95% CI)	Error Rate
≥4 CTCs (I)	0.500	0.986	0.900	0.885	0.760 (0.613–0.908)	0.114
PSA > 25ng/mL (II)	73.680	37.650	0.571	0.878	0.669 (0.508–0.829)	0.163
Gleason sum = 9 pts (III)	0.211	0.929	0.400	0.840	0.697 (0.570–0.825)	0.202
cT > 2c (IV)	0.737	0.595	0.292	0.909	0.666 (0.534–0.798)	0.379
D’Amico criteria = 3	0.316	0.964	0.667	0.862	0.729 (0.593–0.865)	0.155
I + II + III + IV	0.611	0.971	0.846	0.905	0.901 (0.810–0.993)	0.103

AUC—Area Under the Curve.

## Supplementary Materials

The following are available online at https://www.mdpi.com/2072-6694/12/1/160/s1. Table S1: Spearman (*R*) coefficient of correlation between CTC counts determined with various assays, age and clinical characteristics of prostate cancer patients, Table S2: Distribution of clinical characteristics in prostate cancer patients who tested positively (+) or negatively (–) for CTCs with various assays, Table S3: Distribution of patients with distant metastases of prostate cancer who satisfied individual criteria of tested diagnostic algorithm.
