# Supplementary material for: Circulating Tumor Cells as a Marker of Disseminated Disease in Patients with Newly Diagnosed High-Risk Prostate Cancer
Source: Cancers (Basel). 2020 Jan 9;12(1):160. doi: 10.3390/cancers12010160 (PMC7017349; doi:10.3390/cancers12010160)
Supplement: Supplementary file 1 [file cancers-12-00160-s001.pdf]

cancers

MDPI

Article

Circulating Tumor Cells as a Marker of Disseminated Disease in Patients with Newly Diagnosed High-Risk Prostate Cancer

Wojciech A. Cieślikowski, Joanna Budna-Tukan, Monika Świerczewska, Agnieszka Ida, Michał Hrab, Agnieszka Jankowiak, Martine Mazel, Michał Nowicki, Piotr Milecki, Klaus Pantel, Catherine Alix-Panabières, Maciej Zabel and Andrzej Antczak

Table S1. Spearman (R) coefficient of correlation between CTC counts determined with various assays, age and clinical characteristics of prostate cancer patients.

Variable	Cell Collector®	EPISPOT	Cell Search®
Age (years)	$R = -0.069$, $p = 0.488$	$R = -0.026$, $p = 0.799$	$R = 0.057$, $p = 0.597$
PSA (ng/mL)	$R = 0.049$, $p = 0.623$	$R = 0.023$, $p = 0.821$	$R = 0.082$, $p = 0.446$
Gleason sum (pts)	$R = -0.008$, $p = 0.939$	$R = -0.125$, $p = 0.216$	$R = 0.280$, $p = 0.008$
D'Amico criteria (*n*)	$R = 0.004$, $p = 0.966$	$R = -0.071$, $p = 0.482$	$R = 0.190$, $p = 0.078$

Table S2. Distribution of clinical characteristics in prostate cancer patients who tested positively (+) or negatively (–) for CTCs with various assays.

Assay		PSA >20 ng/mL	Gleason sum ≥8 pts	cT >2c
Cell Collector®	(+)	51/60 (85.0%)	16/60 (26.7%)	27/60 (45.0%)
	(–)	30/44 (68.2%)	13/44 (29.5%)	21/43 (48.8%)
	p-value	0.056	0.826	0.841
EPISPOT	(+)	40/52 (76.9%)	11/52 (21.2%)	26/51 (51.0%)
	(–)	37/48 (77.1%)	18/48 (37.5%)	22/48 (45.8%)
	p-value	0.985	0.081	0.689
Cell Search®	(+)	16/21 (76.2%)	11/21 (52.4%)	11/21 (52.4%)
	(–)	56/67 (83/6%)	14/67 (20.9%)	29/66 (43.9%)
	p-value	0.519	0.011	0.617

Table S3. Distribution of patients with distant metastases of prostate cancer who satisfied individual criteria of tested diagnostic algorithm.

Criteria (*n*)	≥4 CTCs (Cell Search®)	PSA > 75 ng/mL	Gleason sum = 9 pts	cT > 2c	Patients (*n*)
3	+	+	−	+	2
3	+	−	+	+	2
2	+	+	−	−	1
2	+	−	+	−	1
2	+	−	−	+	2
2	−	+	−	+	3
2	−	−	+	+	1
2	*n/d*	+	−	+	1
1	+	−	−	−	1
1	−	+	−	−	1
1	−	−	−	+	3
0	−	−	−	−	1

n/d—not determined.
